# Lower Plasma Antioxidant Defense and Heat Shock Proteins Mark Intra‐Amniotic Sludge Ultrasound Finding

**DOI:** 10.1002/jum.70148

**Published:** 2025-12-06

**Authors:** Clarissa Chavez Ortiz Roberto, Lílian Corrêa Costa‐Beber, Lucas Machado Sulzbacher, Jaíne Borges dos Santos, Anna Karolina Kretschmann Florencio de Souza Bagetti, Eduardo Becker, Matias Nunes Frizzo, Thiago Gomes Heck, Mirna Stela Ludwig

**Affiliations:** ^1^ Research Group in Physiology, Postgraduate Program in Integral Attention to Health (PPGAIS‐UNIJUÍ/UNICRUZ) Regional University of Northwestern Rio Grande do Sul State (UNIJUÍ) Ijuí RS Brazil; ^2^ Postgraduate Program in Biological Sciences, Biochemistry Federal University of Rio Grande do Sul (UFRGS) Porto Alegre RS Brazil; ^3^ Fetal Medicine Services Moinhos de Vento Hospital Porto Alegre RS Brazil; ^4^ Postgraduate Program in Mathematical and Computational Modeling (PPGMMC) Regional University of Northwestern Rio Grande do Sul State (UNIJUÍ) Ijuí RS Brazil

**Keywords:** heat shock proteins, intra‐amniotic inflammation, leukocytes, oxidative stress, pregnancy

## Abstract

**Objectives:**

Intra‐amniotic sludge is an independent risk factor for preterm birth. It consists of a biophysical sonographic finding in the amniotic fluid, formed by dense echogenic particles near the cervical internal orifice, due to sterile or infectious inflammatory processes. High‐risk pregnancies are usually marked by oxidative stress, which is one of the main triggers for 70 kDa‐heat shock proteins (HSP70) expression and release. In the blood, eHSP70 marks cellular stress or damage and plays roles in immune‐inflammatory responses. However, the association between sludge, oxidative stress and eHSP70 remained unclear until now. The objective of this study is to evaluate if plasma eHSP70 and redox parameters could distinguish subpopulations with sludge or not.

**Methods:**

We recruited women seeking routine ultrasonography in the second trimester of gestation that met the inclusion criteria. They underwent transvaginal ultrasonography, completed the clinical survey, and provided blood samples for analysis.

**Results:**

Women with sludge exhibited lower antioxidant defense, and eHSP70 levels. They also presented significant changes in the hemogram, suggesting an increase in immune tolerance. In this population, lower HSP70 is associated with a better immunological scenario and proper cervical length to the gestational age.

**Conclusion:**

Blood parameters, oxidative parameters and eHSP70 can indicate intra‐amniotic inflammation.

**Abbreviations**
BHT: butylated hydroxytoluene
BMI: body mass index
CAT: catalase
DAMP: damage‐associated molecular pattern
FICF: Free and Informed Consent Form
HPLC: high‐performance liquid chromatography
HSR: heat shock response
ICMJE: International Committee of Medical Journal Editors
ISUOG: International Society of Ultrasonography in Gynecology and Obstetrics
ROS: reactive oxygen species
SDS: sodium dodecyl sulfate
SOD: superoxide dismutase
TBA: thiobarbituric acid
TBARS: thiobarbituric acid reactive substances
TORCH: toxoplasmosis, rubella, cytomegalovirus, herpes
TVUS: transvaginal ultrasonography

Spontaneous preterm birth affects approximately 5–13% of pregnancies and stands as a leading contributor to perinatal morbidity and mortality.^1^ One significant risk factor for this outcome is the presence of intra‐amniotic sludge,^2^ which is identified through sonography as dense, fluctuating, and aggregated echogenic particles in the amniotic fluid, situated near the internal orifice.^3^ These particles can correspond to vernix, meconium, blood, and inflammatory material associated with intraamniotic sterile or infectious inflammation,^4^, ^5^ or to desquamated skin cells.^3^ Sludge finding is associated with short cervix, which enhances the odds to preterm delivery.^5^

Adverse gestational outcomes are associated with oxidative stress, inflammation and infection by mechanisms that are not fully understood yet.^3^ During gestation, there is a physiological increase in metabolism, with a huge demand for O_2_ oxidation and ATP generation, to allow fetal growth and development.^6^ There is also a set of adaptations in the immune system to tolerate fetus growth, that include a slight decrease in polymorphonuclear leukocytes activity, and an increase in the number of total leukocytes^7^ from the second trimester onwards. When the mother's antioxidant defense does not follow the rate of metabolism's increase, she can develop oxidative stress.^8^ Oxidative stress triggers aging, carcinogenesis and chronic inflammatory diseases. Moreover, during gestation, the existence of an oxidative milieu can promote immune activation, which is associated with complications and adverse gestational outcomes.^9^

Oxidative stress and immune activation are powerful triggers to heat shock response (HSR), through the activation of the 70 kDa—Heat Shock Protein (HSP70).^10^ HSP70 presents important chaperone and anti‐inflammatory roles within the intracellular medium (iHSP70), but opposite functions when released in the extracellular medium (eHSP70).^11^ In blood, eHSP70 can mediate inflammation by triggering innate immunity and signaling as molecular patterns of cellular damage or alarmins.^12^, ^13^ In this medium, they can regulate and interact with leukocytes and define the immune and inflammatory profile during the infection or disease.^14^ However, HSP70 modulation during healthy or sludge gestation is still unclear.

In this study, we intended to characterize immune, oxidative and heat shock parameters in blood from pregnant women with sludge. We hypothesized that eHSP70 and redox parameters in pregnant women's plasma could distinguish the subpopulations of high‐risk pregnancies.

## Material and Methods

### Ethical Aspects

This was a prospective cohort study, previously approved by the Research Ethics Committee from UNICRUZ (Approval n° 3.255.646, Annex 01) and from the Municipal Health Department of the municipality of Ijuí, Brazil The study was carried out in accordance with the World Medical Association Declaration of Helsinki and followed the International Committee of Medical Journal Editors (ICMJE) recommendations. The privacy rights of human subjects were observed, and informed consent was obtained for experimentation with human subjects.

### Study Design

**Figure 1:**
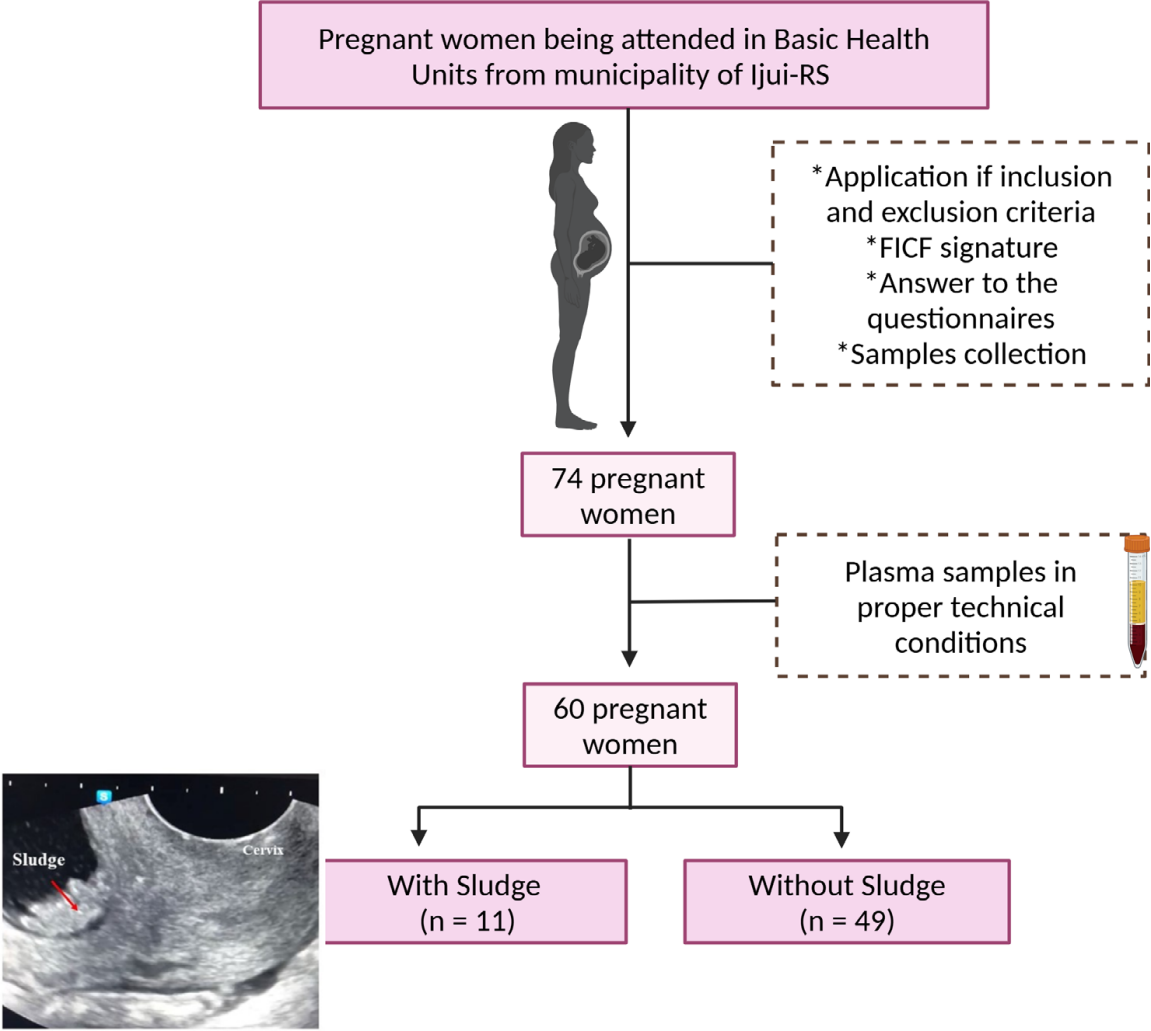
Experimental design: women's inclusion and separation into the categories for comparison.

The study was carried out in Basic Health Units from the municipality of Ijuí (Brazil) and in a private clinic of ultrasonography in the same locality. Women looking for health services and routine ultrasonography in the second trimester of gestation that fulfilled the inclusion criteria were invited to participate in the study (Figure 1). The following criteria were applied: to have single and spontaneous gestation, absence of clinical symptoms suggestive of a high‐risk pregnancy or underlying disease and be between 15 and 27 weeks of pregnancy. Women who had undergone assisted fertilization, had any infectious disease, such as syphilis, toxoplasmosis, rubella, cytomegalovirus, herpes (TORCH), acquired immunodeficiency syndrome (HIV), hepatitis, or an active autoimmune disease such as systemic lupus erythematosus, rheumatoid arthritis, antiphospholipid syndrome; were on antibiotics, corticosteroids, or anti‐inflammatory drugs on the day of the examination; had clinical symptoms or suspected infection; had active vaginal bleeding; or had uterine contractions were excluded from the analysis.

A total of 74 women signed the Free and Informed Consent Form (FICF), had obstetric and transvaginal US, collected biologic samples, and answered the clinical questionnaire. For the laboratorial analysis, samples with hemolysis were excluded, ending the experimental design with a total of 60 pregnant women. For comparison, the subjects were classified into “sludge” and “normal” according to the diagnosis made in the second gestational trimester (Figure 1).

### Data Collection

#### Demographic Data

The pregnant women answered a survey based on screening models for prematurity and preeclampsia recommended by the International Society of Ultrasonography in Gynecology and Obstetrics (ISUOG), in which the sociodemographic profile of the pregnant women was compiled; the clinical and obstetric history that included previous miscarriages and preterm labor, parity, active diseases, previous, or other comorbidities in previous pregnancies; and researched other risk factors for prematurity such as ethnicity, age, smoking, use of drugs, medicine, and uterine malformations (Tables S1 and S2).

#### Obstetric Ultrasound

Obstetric ultrasound was performed by suprapubic evaluation, using a convex transducer of 3–5 Hz and gel on the abdomen of the pregnant woman, to calculate the fetal weight according to the Hadlock Table 3, based on the measurements of the biparietal diameter, head circumference, abdominal circumference, and fetal femur length; evaluation of fetal morphology; amount of amniotic fluid; and position of the placenta.

#### Endovaginal Ultrasound

**Figure 2:**
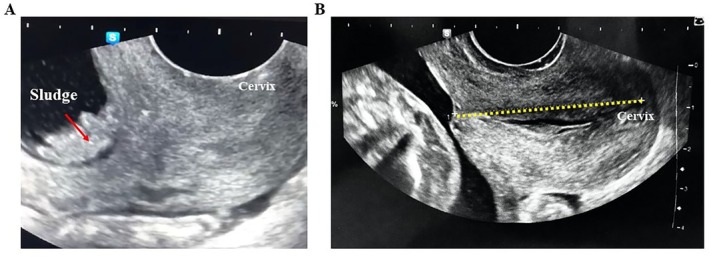
**A**, Sludge. A 21‐year‐old pregnant women, 16 weeks of pregnancy. Transvaginal US performed with the endocavitary transducer of the SAMSUNG H60 device. **B**, Cervix measurement. A 26‐year‐old pregnant women, 24 weeks of pregnancy. Transvaginal US performed with the endocavitary transducer of the SAMSUNG H60 device.

It was performed to measure the uterine cervix (Figure 2) according to the standard technique,^15^ introducing the endocavitary probe into the vagina (frequency of 5.0–9.0 MHz), with a condom and gel, directing it to the anterior fornix, without exerting pressure against the cervix, since compression can artificially increase the cervical measurement. The endocervical mucosa, which can be hyperechogenic or hypoechoic in relation to the cervix, was used as a guide for the location of the internal (OI) and the external orifice of the cervix (LE), to avoid confusion with the lower segment of the uterus. The reference limits for the measurement were the V‐shaped notch in the OI and the more echogenic triangular area in the LE, with measurement of the linear distance between them. In curved necks, the measurement was also performed in these parameters in a line; the lowest measurement that was verified in the observation period was recorded.

#### Intra‐Amniotic Sludge Diagnosis

Sludge was evaluated through transvaginal ultrasonography (TVUS), and considered positive in the presence of dense, aggregated, floating echogenic particles located near the internal orifice of the cervix^16^ (Figure 2). When these criteria were obeyed, the diagnosis was confirmed through photos and/or videos by a second sonographer.

#### eHSP70 Measurement

eHSP72 levels were measured in plasma samples by a highly sensitive enzyme‐linked immunosorbent assay, with sensibility to 7 pg/mL. We used an HSPA1A‐specific HSP72 ELISA Kit (ENZO Life Sciences, ENZ‐KIT‐101) according to the manufacturer's recommendations. A standard curve was constructed from known dilutions of HSP72 recombinant protein to allow a quantitative assessment of eHSP72 plasma concentration. Quantification was done using a microplate reader (Mindray MR‐96A) at 495 nm.

#### Redox Analysis

Lipoperoxidation was determined through the thiobarbituric acid reactive substances method (TBARS).^17^ Briefly, plasma was incubated with thiobarbituric acid (TBA, 0.6%), butylated hydroxytoluene (BHT, 10 mM), phosphoric acid (H_3_PO_4_, 1%), and sodium dodecyl sulfate (SDS, 8.1%) for 60 min at 100°C. After this, tubes were centrifuged, the supernatant collected, and the absorbance verified in a plate reader (DR‐200BS model, Kasuaki, PR, Brazil) at 505 nm. The MDA standard was prepared from 1.1.3.3‐Tetramethoxypropane (points from 0.0005–0.016 mg/mL).

Antioxidant defense was also evaluated through superoxide dismutase (SOD) and catalase (CAT) activities. SOD was determined through the inhibition of pyrogallol auto‐oxidation, at 420 nm.^18^ CAT was evaluated through hydrogen peroxide decomposition, at 240 nm.^19^ We followed the methods previously detailed.^20^

#### Hematological Analysis

Hematological analysis was performed automatically using the ABX Micros 60 hematology analyzer (HORIBA). With this equipment, we obtained parameters for red, white, and platelet series, as follows. Red series: total red blood cell count, hemoglobin, hematocrit, mean corpuscular volume, mean corpuscular hemoglobin, mean corpuscular hemoglobin concentration, and red cell distribution width. White series: total leukocyte count, differential leukocyte count, and platelet count. We calculated the monocyte‐to‐lymphocyte ratio, neutrophil‐to‐lymphocyte and platelet to lymphocyte ratio based on the absolute count. We also included systemic inflammatory response index SIRI=Neutrophils×Monocytes/Lymphocytes, and aggregate index of systemic inflammation AISI=Neutrophils×Platelets×Monocytes/Lymphocytes, as previously detailed.^21^ Besides, glycated hemoglobin was measured in whole blood aliquots by high‐performance liquid chromatography (HPLC).

### Statistical Analysis

Data were expressed as mean ± SD. Data normality was verified through the Kolmogorov–Smirnov test. Then, the Mann–Whitney test was used to compare non‐parametric data, and the Student t‐test was used for parametric data. Two‐way ANOVA was also used when pertinent. The Fischer exact test was used to compare categorical variables. Pearson and Spearman correlations were also used to test the association between variables. All statistical analyses were carried out in GraphPad Prism, 10.03. Significance was defined using *p* < .05.

## Results

### Clinical Characteristics of the Study Population

In our study, 18.3% of the pregnant women were diagnosed with sludge (11/60), which was identified by the presence of dense, aggregated, buoyant particles located near the internal orifice of the cervix,^16^ as demonstrated in Figure 2.

Women with and without amniotic fluid sludge had similar age, body mass index (BMI), glycated hemoglobin, systolic and diastolic blood pressure. The sludge group tended to exhibit a smaller cervix (*p* = .058). Only 1 woman from the ‘normal’ group and another from the “sludge” group presented abnormal measurements (2.5 and 2.2 cm, respectively). The fetal weight was not different among groups and was considered adequate for gestational age in all patients (Table 1).

**Table 1:** General and Gestational Parameters

Parameters	Normal (n = 49)	Sludge (n = 11)	*p* Value
General parameters			
Age (years)	25.78 ± 5.705	24.82 ± 6.322	.623
BMI (kg/m^2^)	35.41 ± 50.43	47.23 ± 71.05	.518
Glycated hemoglobin (%)	5.0 ± 0.3	5.0 ± 0.2	.546
Systolic blood pressure (mmHg)	113.2 ± 13.3	116.8 ± 8.2	.468
Diastolic blood pressure (mmHg)	70.45 ± 11.5	73.64 ± 8.1	.504
Gestational parameters			
Gestational age (weeks)	19. 9 ± 3.2	18.2 ± 3.6	.120
Cervical length (cm)	3.6 ± 0.5	3.2 ± 0.5	.058
Fetal weight (g)	396. 1 ± 227.0	305.1 ± 224.7	.233
Biparietal diameter (cm)	4.647 ± 1.0	4.191 ± 1.2	.199
Head circumference (cm)	17.61 ± 3.8	15.59 ± 4.4	.126
Fetal abdominal circumference (cm)	14.98 ± 3.6	13.13 ± 4.1	.140
Length of the femur (cm)	3.276 ± 1.0	2.845 ± 1.0	.186

Data expressed in mean ± SD and compared through student's *t*‐test.

### Sludge Diagnosis Is Marked by Antioxidant Defense Imbalance

**Figure 3:**
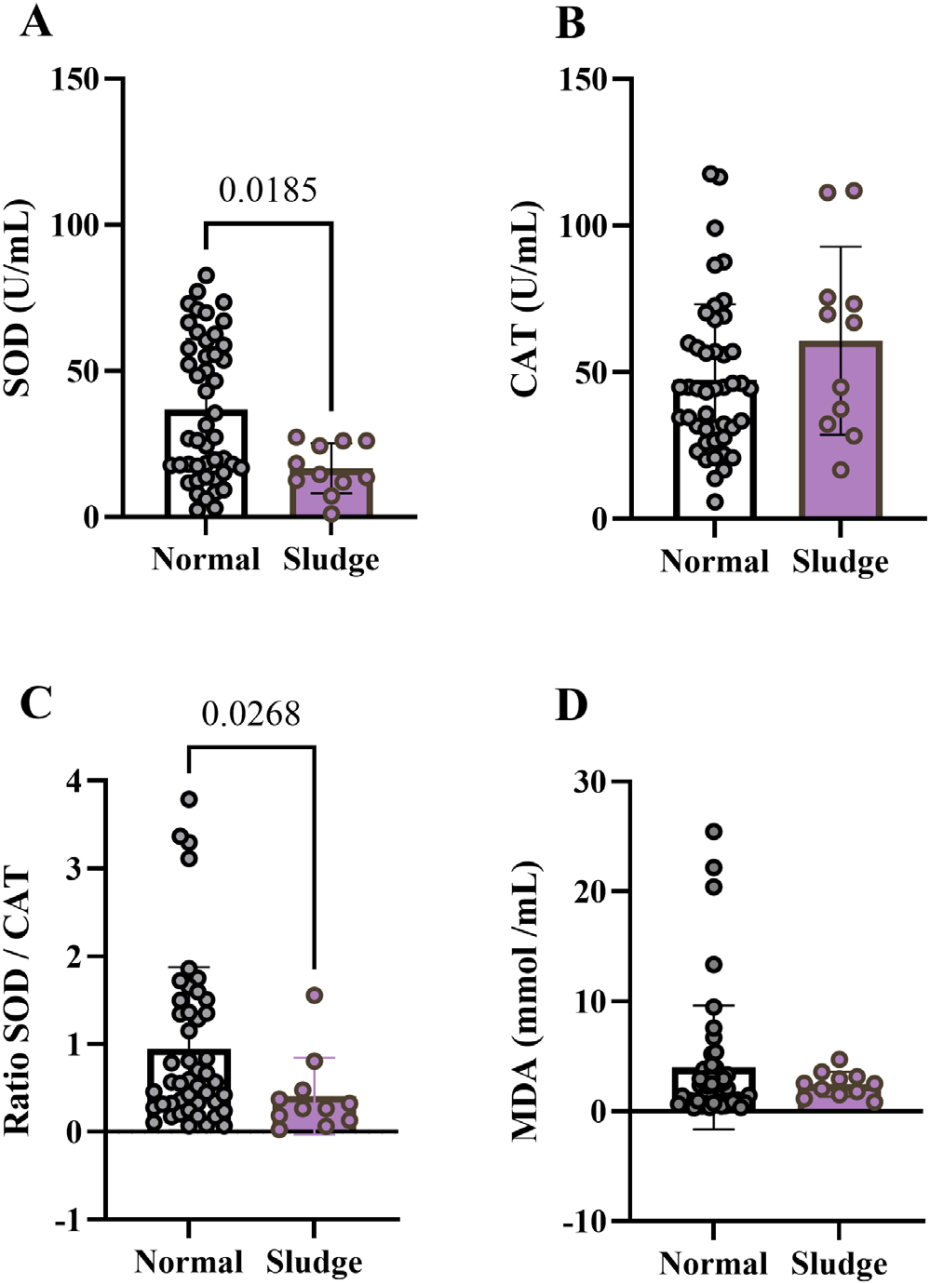
Diagnosis of sludge is marked by oxidative stress. **A**, SOD activity. **B**, CAT activity. **C**, SOD to CAT ratio. **D**, Lipoperoxidation (MDA levels). Data presented as mean ± SD and compared by Mann–Whitney test. N = 11 for sludge group, and N = 49 for normal group.

Pregnant women diagnosed with sludge presented lower SOD antioxidant activity (Figure 3A). CAT activity did not differ (Figure 3B), resulting in a reduced SOD to CAT ratio in the sludge group (Figure 3C). These results may indicate an imbalance between antioxidant enzymes, which could either promote oxidative stress or simply reflect a decreased demand for antioxidant defenses. Supporting the second hypothesis, lipoperoxidation levels did not differ among the groups (Figure 3D).

Sludge can be derived from sterile or infectious oxidative and inflammatory processes. Both can be marked by leukocyte subtypes accounts, which are the main involved in reactive oxygen species (ROS) generation within the vessels. Given the depletion of SOD antioxidant defense verified in the sludge group, we investigated if it could be reflected in hematological parameters. Indeed, women with sludge presented more lymphocytes and monocytes than their respective controls. Curiously, the neutrophil‐to‐lymphocyte ratio, a measurement associated with disordered inflammation and immunity and overall mortality,^22^ was reduced in the sludge group. None of the other inflammatory and hematocrit parameters were altered (Table 2).

**Table 2:** Hematological Parameters According to the Occurrence of Sludge

Parameters	Normal (n = 47)	Sludge (n = 11)	*p* Value
Erythrocytes (10^6^/mm^3^)	4.0 ± 0.4	4.2 ± 0.6	.238
Total leukocytes (10^3^/mm^3^)	9.4 ± 2.6	9.3 ± 2.1	.917
Lymphocytes (10^3^/mm^3^)	1.8 ± 499.7	2.2 ± 584.7	.040*
Neutrophils (10^3^/mm^3^)	6.8 ± 2.1	6.3 ± 1.6	.451
Monocytes (cells/mm^3^)	497.7 ± 146.1	619.1 ± 188.4	.026*
Eosinophils (cells /mm^3^)	162.0 ± 122.0	170.6 ± 138.2	.839
Basophils (cells /mm^3^)	18.6 ± 11.5	19.6 ± 7.3	.801
Platelets (10^3^/mm^3^)	218.0 ± 498.2	239.4 ± 499.1	.208
Hemoglobin (Hb) (g/dL)	12.1 ± 0.9	12.3 ± 1.3	.566
Hematocrit (%)	35.7 ± 2.5	36.0 ± 3.8	.711
Mean corpuscular volume (Fl)	89.5 ± 3.2	87.1 ± 5.1	.054
Mean corpuscular hemoglobin (g)	30.5 ± 1.2	29.9 ± 2.0	.161
Mean corpuscular hemoglobin concentration (%)	34.1 ± 0.7	34.3 ± 0.9	.514
Red cell distribution width (%)	13.2 ± 0.6	13.1 ± 0.8	.429
Neutrophil‐to‐lymphocyte ratio	3.9 ± 1.3	3.0 ± 1.0	.039*
Monocyte‐to‐lymphocyte ratio	0.287 ± 0.099	0.296 ± 0.114	.792
Platelet‐to‐lymphocyte ratio	128.8 ± 45.3	114.2 ± 31.0	.319
SIRI	1983 ± 999.3	1882 ± 849.4	.759
AISI	453905681 ± 275099387	450727273 ± 229062913	.971

Data expressed in mean ± SD and compared through student's *t*‐test. * P < 0.05.

### eHSP70 Levels Are Inversely Associated with Cervical Length in Pregnant Women with Sludge

**Figure 4:**
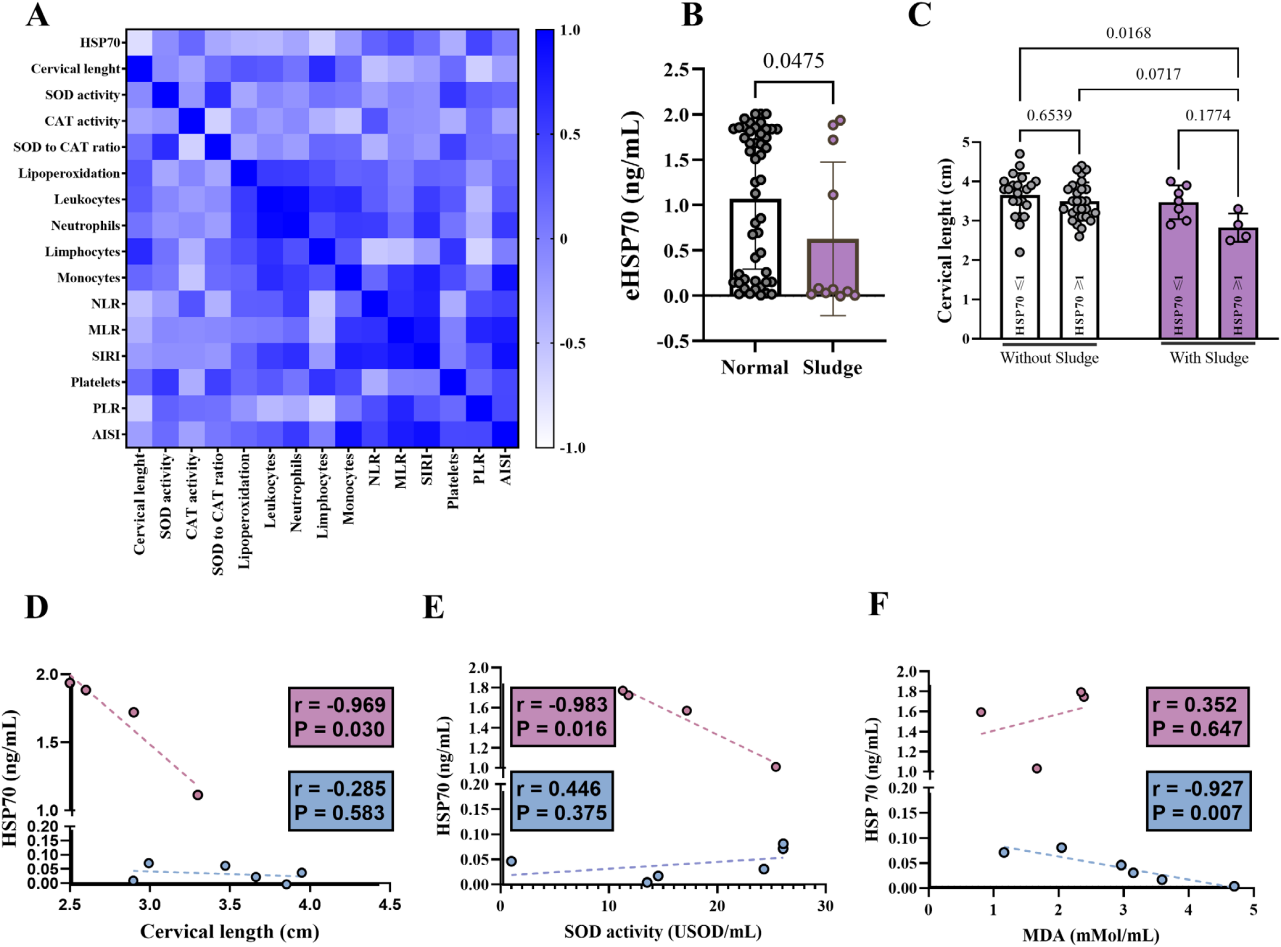
Diagnosis of sludge is marked by lower HSP70 levels, which are inversely associated with cervix size, SOD activity, and lipoperoxidation. **A**, Heatmap showing correlations between parameters. **B**, HSP70 levels among pregnant women with or without sludge (Mann–Whitney). **C**, Cervix length compared among pregnant women with and without sludge according to the HSP70 levels (2‐way ANOVA: HSP70: F_1,56_ = 5.572; *p* = .021. Sludge: F_1,56_ = 6.167; *p* = .016. Interaction between factors: F_1,56_ = 1.942; *p* = .169). **D**, Pearson's correlation between HSP70 levels and cervix size. **E**, Association between HSP70 levels and SOD activity. **F**, Pearson's correlation between HSP70 levels and lipoperoxidation.

Given that sludge was already related to inflammation, we measured eHSP70 levels as indicative of the immune and inflammatory activation. Curiously, women with sludge presented lower eHSP70 levels (0.63 ± 0.85 ɲg/mL) compared to their respective controls (1.07 ± 0.78 ɲg/mL) (Figure 4B). Among women with sludge, eHSP70 was inversely correlated with cervical length (*r* = −0.734; *p* = .015*) and lymphocyte numbers (*r* = −0.619; *p* = .056) (Figure 4A).

A previous study focusing on cervical length showed that eHSP70 is lower in patients with a short cervix.^23^ Short cervical length is a physical feature closely related to preterm birth^1^ and small fetus for the gestational age.^24^ Taking this into consideration, we observed that our sludge population was very heterogeneous regarding the eHSP70 levels. Then, we stratified the control and sludge populations in eHSP70 lower than or equal to 1, and higher than 1 (HSP70 ≤1 and HSP70 >1), wondering if it could be associated with cervical length in the sludge context. We conducted a 2‐way ANOVA, which considers the presence of sludge and the eHSP70 levels as independent factors (row versus column). According to the test, patients with sludge presented significantly shorter cervical length, which was exacerbated when the diagnosis was combined with eHSP70 levels higher than 1 (2‐way ANOVA: sludge: *p* = .016; eHSP70: *p* = .0218; Interaction between factors: *p* = .169) (Figure 4C), supporting both as risk factors for short cervix.

We performed additional correlational analysis between pertinent parameters within both sludge subpopulations (HSP70 ≤1 and HSP70 >1). eHSP70 was inversely correlated with cervical length (Figure 4D) and SOD activity (Figure 4E) in the HSP70 >1 subpopulation. It also exhibited an inverse association with lipoperoxidation levels in the HSP70 ≤1 population (Figure 4F). It did not correlate with other inflammatory variables, except for eosinophils' account in the HSP70 >1 population (Table 3). Together, these results suggest that the lower eHSP70 levels mark an immunotolerance in women with sludge.

**Table 3:** HSP70 Association with Inflammatory Markers

Parameters	HSP70 ≤1.0 (n = 7)		HSP70 >1.0 (n = 4)	
	*R* Value	*p* Value	*R* Value	*p* Value
Total leukocytes (10^3^/mm^3^)	0.003	.994	−0.340	.659
Lymphocytes (10^3^/mm^3^)	−0.022	.965	−0.439	.560
Neutrophils (10^3^/mm^3^)	−0070	.893	−0.256	.743
Monocytes (cells/mm^3^)	0.486	.328	−0.316	.683
Eosinophils (cells /mm^3^)	0.084	.873	−0.963	.036*
Basophils (cells /mm^3^)	0.587	.220	0.730	.269
Platelets (10^3^/mm^3^)	0.292	.573	0.083	.918
Neutrophil‐to‐lymphocyte ratio	−0.209	.690	0.056	.943
Monocyte‐to‐lymphocyte ratio	0.495	.318	0.090	.909
Platelet‐to‐lymphocyte ratio	0.263	.613	0.384	.615
SIRI	0.282	.587	−0.133	.866
AISI	0.519	.290	−0.055	.944

* P < 0.05, Pearson or Sperman correlation.

## Discussion

In our study, intra‐amniotic sludge was marked by a decreased antioxidant defense and lower eHSP70 levels. It was also characterized by a lower neutrophils to lymphocytes ratio, suggesting a weak immune and inflammatory response, and immune tolerance to the fetus. The tendency to present shorter cervical length among women diagnosed with sludge was distinguished through eHSP70 levels. Lower eHSP70 levels marked a longer cervix, higher eosinophils account, SOD antioxidant defense and lipoperoxidation levels in pregnant women with sludge. Altogether, our data show that changes in hemogram and eHSP70 could be indicative of intra‐amniotic inflammation.

During pregnancy, the metabolic activity within the fetal‐placenta compartment^25^ increases the oxidative metabolism and ROS generation.^26^ This whole scenario demands antioxidant defense to neutralize ROS and to prevent them from spreading oxidative damage. An impairment in the antioxidant defense, such as SOD activity can mark adverse gestational outcomes.^27^ Indeed, compared to the other pregnant women, the group diagnosed with sludge exhibited lower SOD activity. SOD is the first antioxidant defense against superoxide anion (O_2_^−^),^28^ generating hydrogen peroxide (H_2_O_2_), which is a substrate to CAT and glutathione activities. Therefore, an independent overactivation of SOD (high SOD‐to‐CAT ratio) would result in an increase in H_2_O_2_ and hydroxyl radicals (OH^−^) generation through Fenton reactions, and an exacerbation in the oxidative injury. Interestingly, women with sludge had a lower SOD‐to‐CAT ratio and similar lipoperoxidation than other pregnant women, suggesting a lower requirement for antioxidant defense or oxidative tolerance.

Gestation is marked by a gradual change in the immune system.^29^ It switches toward humoral immunity, while the cell‐mediated (cytotoxic) immune response is suppressed.^7^, ^29^ This whole change aims to protect the fetus from the cellular cytotoxic response,^29^ but can jeopardize the mother's ability to deal with opportunistic infections. We found that monocytes and lymphocytes numbers were higher in the sludge group, but the neutrophil‐to‐lymphocyte ratio was lower, indicating a potential shift in the immune response toward a regulatory profile rather than an inflammatory one.^22^, ^30^ Sludge is characterized by sterile or infectious biofilm, that decreases the efficacy of the mother's immune system to recognize inflammatory agents,^31^, ^32^ which could explain these results obtained from the mother's leukogram. Additionally, AISI and SIRI, other good markers of inflammation,^21^ were not affected by sludge diagnosis. Therefore, the lower neutrophil‐to‐lymphocyte and SOD‐to‐CAT ratios compared to other pregnant women could reinforce a deficit in the immune system from women with sludge and suggest immune tolerance.

An additional mechanism for maintaining immunological tolerance toward the fetus involves the physiological reduction of eHSP70 levels.^33^ Within the intracellular medium, iHSP70 has anti‐inflammatory activity, through the stabilization of the NF‐κB cytoplasmic complex, and consequent blockade of inflammatory cytokine transcription.^11^ However, in the extracellular medium, eHSP70 acts as a damage‐associated molecular pattern (DAMP) and can bind to cell surface receptors (eg, Toll‐like receptors 2 and 4) and command NF‐κB activation.^11^ As it is observed under severe cell stress, experimental studies indicate that pathological conditions, such as hypertension, asthma, diabetes during pregnancy, or even preterm labor, are associated with elevated eHSP70 levels compared to normal pregnancies,^33^, ^34^ supporting the existence of a dangerous and inflammatory microenvironment. Given the observed changes in the leukocyte subpopulation profiling in the sludge patients, and that HSP70 expression and release can be induced by stress,^35^ we investigated whether sludge could affect its levels. Interestingly, we found lower eHSP70 levels in women diagnosed with sludge, which were inversely correlated with lymphocyte numbers. Considering that sludge is marked by intra‐amniotic inflammation,^4^ and that it could impair immune tolerance, the decrease in the neutrophil‐to‐lymphocyte ratio could be inferred as a possible link to immune deregulation, specifically to control eHSP70 levels. In this sense, the eHSP70 levels could be attenuated by anti‐HSP70 auto‐antibodies, as a protective attempt to keep the tolerance to the fetus,^33^ given that its presence in the amniotic cavity, is associated with neonatal comorbidity.^12^

The imbalance between anti‐/and pro‐inflammatory mediators in the cervical‐vaginal milieu weakens cervical integrity, shortens it,^36^ and enhances the susceptibility to adverse gestational outcomes.^1^, ^24^ Here, we found that eHSP70 levels were inversely associated with cervical length in the sludge population. After careful stratification, we observed that women with HSP70 >1 exhibited smaller cervix than their counterparts, and had their cervical lengths negatively correlated with HSP70 levels. HSP70 levels were also correlated with redox parameters, reflecting their immune roles, such as T cells, natural killers and macrophages activators.^37^ Together these results supported that among the population diagnosed with sludge, lower HSP70 levels are associated with a proper cervical length to the gestational period. Finally, considering that sludge can be associated with infectious or sterile inflammation,^4^ whose resolution is jeopardized by excessive or weak cellular response, it is plausible that a certain level of eHSP70 might still be necessary to keep the proper anti‐/pro‐inflammatory balance in sludge patients, while the increase in it could jeopardize the natural gestational course.

Literature still lacks data on the oscillation of eHSP70 levels during different pregnancies. However, to our knowledge, our study is the first to show that sludge ultrasound findings are marked by a decrease in systemic antioxidant defense and eHSP70 levels, and leukogram changes that support an enhancement of immune tolerance. Our results reflect changes in the mother's blood during the second trimester of pregnancy, which is the best period for fetal assessment,^38^ able to detect the mother's glycaemia, blood pressure and the infections, including sludge, and predict high‐risk births.^39^ We found that the lower eHSP70 levels in sludge may reflect a protective attempt to prevent an exacerbated immunological reaction that could impact fetal survival and jeopardize cervical structure. We highlight that the associations could be stronger, but suggestive clinical symptoms of a high‐risk pregnancy or underlying disease, or suspected infection and active vaginal bleeding were used as exclusion criteria. Finally, our data support blood parameters as indicative of changes in sludge, but the mechanisms underlying this association remain to be further studied.

## Conclusion

Intra‐amniotic sludge is marked by lower plasma antioxidant defense and eHSP70 levels. Among this population, lower HSP70 levels are associated with a better immunological scenario and proper cervical length to the gestational age. Finally, our data support that blood parameters can indicate intra‐amniotic inflammation.

## Supporting information


**Table S1.** Sociodemographic profile.
**Table S2.** Obstetric history of pregnant women according to the occurrence of Sludge.

## Data Availability

The data that support the findings of this study are available from the corresponding author upon reasonable request.
