# Supplementary material for: Lower Plasma Antioxidant Defense and Heat Shock Proteins Mark Intra‐Amniotic Sludge Ultrasound Finding
Source: J Ultrasound Med. 2025 Dec 6;45(5):1083–94. doi: 10.1002/jum.70148 (PMC13053630; doi:10.1002/jum.70148)
Supplement: Supplementary file 1 — Table S1. Sociodemographic profile. Table S2. Obstetric history of pregnant women according to the occurrence of Sludge. [file JUM-45-1083-s001.docx]

**SUPPLEMENTARY MATERIAL**

**Tabela S1**: Sociodemographic profile.

| **Features** | | **Population (n = 60)** |
| --- | --- | --- |
| Maternal age | Mean ± SD | 25.6 ± 5.7 |
|  | Median | 23.5 |
|  | IL – SL | 17 – 40 |
| Marital status - n (%) | With partner | 12 (20) |
|  | Without partner | 48 (80) |
| Schooling - n (%) | High School | 49 (81.6) |
|  | Graduation | 11 (19.4) |
| Ethnicity - n (%) | White | 38 (63.3) |
|  | Black | 16 (26.6) |
|  | Brown | 6 (10) |
| Minimum wage - n (%) | Between 0 and 2 p/month | 54 (90) |
|  | Between 2 and 5 / mês | 6 (10) |

IL = Inferior limit; SL = Superior limit.

**Table S2:** Obstetric history of pregnant women according to the occurrence of Sludge.

| **Features** | | **Without S*ludge* (n=49)** | **With S*ludge* (n=11)** | ***P Value*** |
| --- | --- | --- | --- | --- |
| Parity- n (%) | Multiparous | 31 (63.3) | 6 (54.5) | 0.417^a^ |
|  | Nulliparous | 18 (36.7) | 5 (45.5) |  |
| Previous preterm labor - n (%) | No | 43 (87.8) | 10 (90.9) | 0.621^a^ |
|  | Yes | 6 (12.2) | 1 (9.1) |  |
| Gestational age at delivery – weeks (mean ± SD) | | 38.33 ± 2.47 | 37.45 ± 3.04 | 0.3125^b^ |
| Total | | 49 (81.7) | 11 (18.3) |  |

^a^ Fischer test; ^b^ Student t-test
